# Emergency Department Boarding, Crowding, and Error

**DOI:** 10.1016/j.acepjo.2025.100169

**Published:** 2025-05-19

**Authors:** Joshua Kolikof, Daniel Shaw, Bryan Stenson, Lakshman Balaji, Anne Grossestreuer, David Chiu

**Affiliations:** Department of Emergency Medicine, Beth Israel Deaconess Medical Center, Boston, Massachusetts, USA

**Keywords:** boarding, crowding, quality improvement, error

## Abstract

**Objectives:**

Emergency department (ED) crowding and boarding have become a public health emergency. Independently, each is associated with morbidity and mortality, but what remains to be elucidated is whether there is an association between these 2 instances and a departmental error. Our objective is to examine adjudicated error as it relates to these 2 instances.

**Methods:**

We performed a retrospective cohort study, analyzing every patient encounter from July 1, 2018 to June 30, 2023 and queried for the presence and absence of an error. We calculated incident rate ratios and controlled for the patient's age, gender, Emergency Severity Index (ESI) level, the ED work score (a surrogate measure of crowding), and ED crowding surge capacity activation. Our primary exposures were crowding and boarding, and our outcome of interest was the presence of error.

**Results:**

Of 250,049 patient encounters, an error rate of 500/100,000 was observed, and there was an increase in both boarding and ED volume. There was a higher likelihood of error with patients whose status was boarding in the ED (adjusted incidence-rate ratios [aIRR] 1.60 [95% CI 1.42-1.82]) and who had higher acuity (ESI 1 IRR 2.9 [95% CI 2.4-3.5], and ESI 2 IRR 1.5 [95% CI 1.3-1.7]) when compared with encounters where no error occurred. There was a lower likelihood of error with a higher ED work score (aIRR 0.81 [95% CI 1.03-1.47]).

**Conclusion:**

In our retrospective cohort study of all ED encounters over the past 5 years, ED crowding and boarding increased but did not appear to portend a higher likelihood of an error. However, higher acuity patients, and those who were themselves boarders, had an increased likelihood of an error in their care.

**The Bottom Line**

Emergency department crowding and boarding are serious threats to the delivery of safe emergency care. This study examined whether crowding and boarding were related to the likelihood of an error occurring. In general crowding and boarding were not associated with a higher likelihood of an error. However, individuals who were more ill or whom themselves were boarding in the emergency department were 1.5 times and 1.6 times more likely to experience an error in their care, respectively.

## 1 Introduction

### 1.1 Background

Emergency department (ED) crowding and boarding are a national crisis^1^ that are directly and indirectly associated with patient mortality and morbidity.^2^ ED crowding is a mismatch between resources necessary to provide safe and appropriate emergency care relative to the volume of patients being evaluated and treated within the ED or the hospital.^3^ This can occur due to a lack of inpatient capacity, inpatient or ED staffing, or other downstream effects.^4^^,^^5^ It is strongly associated with poor patient outcomes and has risen to a national emergency in recent years.^6^ ED boarding is the status of hospitalized patients remaining in the ED while awaiting an inpatient bed for ≥120 minutes.^7^ It, too, is associated with mortality, morbidity, and patient harm.^2^^,^^8^^,^^9^ These 2 circumstances are distinct entities but are often interdependent and result from systemic issues.

Various operational metrics are used to report crowding, such as National Emergency Department Overcrowding Study^10^ and the Emergency Department Work Index,^11^ and routine metrics, such as length of stay, waiting room volume, ED occupancy, boarding time, number of boarders, and waiting room census.^12^ The latter, singular metrics, are surrogate measures that do not provide more complex information regarding ED crowding. A metric that is easily calculated is the work score.^13^^,^^14^ It is calculated based on the number of patients in the waiting room, reversed Emergency Severity Index (ESI), the number of nurses, the number of boarders, and the number of ED treatment areas. This is readily captured minute to minute and does not require information outside of data gathered from the ED, such as intensive care unit capacity. As such, the work score is a helpful tool to gauge crowding, with higher values corresponding to increased crowding.

Although crowding and boarding have been issues for decades, there has been work to mitigate the impact of policy changes that affect ED flow. In particular, when ambulance diversion was outlawed in Massachusetts, legislators correctly identified the need for a mechanism by which hospitals could respond to crowding events.^15^^,^^16^ Code help is a Massachusetts policy intended to move all admitted patients out of the ED within 30 minutes of activation, and is active when the ED is unable to care for existing patients in licensed treatment areas, or there is the inability to accept new patients into those areas due to boarding.^17^

The policy of surge capacity, or “code help” status, enables multiple hospital mechanisms to be activated, whereby ED boarding patients are moved to available hallway spots within the inpatient wards to decompress the ED and alleviate crowding. The status of the ED is either code help clear, alert, or active, with the latter initiating the mechanisms above.^18^ At our institution, an alert level 1 is when ≥5 patients are boarding, an alert level 2 is when either ≥10 patients are boarding or ≥63 are registered awaiting evaluation in the ED. A critical care alert is when ≥4 intensive care unit (ICU) patients are boarding, or a single ICU patient has a bed requested for >6 hours. Any alert level 2 criteria must be met and superseded for activation to proceed. However, there are only a finite number of inpatient hallway spots. In ideal situations, this helps with length of stay and ED boarding times, but its impact on recent crowding has yet to be elucidated.^15^

Although previous work on the topic of error and boarding/crowding has demonstrated an association with boarding/crowding and error as it relates to physician experience level,^19^ what has yet to be directly studied is the likelihood of errors during profound crowding and boarding events, which have been increasing in frequency throughout the nation.^20^ Error is defined broadly by the Institute of Medicine as the failure of a planned action, the use of a wrong plan, or the deviation from standard and accepted practice, which may or may not result in an adverse event.^21^^,^^22^

At our institution, error is adjudicated after a thorough review of the timeline of patient care, events surrounding the error, discussion with physicians and ancillary staff involved, and after a thorough review by peers at a quality assurance committee meeting.^23^ All 72-hour returns, floor-to-ICU admissions within 24 hours, event flags, patient complaints, reported incident flags by any staff member, and adverse events are reviewed by the committee for possible errors.^23^ This information is automatically generated in the case of return visits and floor-to-ICU admissions. When there is an adverse event or when there is the possibility of a deviation in care, any staff member within the department or external to the department can flag it for review within our quality assurance apparatus. The committee can adjudicate any of the following:

• “No error, judgement calls that the reviewer may not have made but can accept; with no apparent consequences.
• Possible errors in care of little consequence that did not compromise care in any appreciable way.
• Moderate errors with resulting consequences that had the potential to compromise care, but which did not appear to compromise care.
• Moderate errors with resulting consequences that may have compromised care.
• Major errors that with consequences that compromised care but where the overall care was within the standard of care.
• Major errors that resulted in compromised care and which violated the standard of care.
• Major errors that grossly violated the standard of care.”^23^

### 1.2 Importance

As boarding and crowding increase potentially posing dangers to the safe delivery of emergency care, the threat of an error is ever present. Few studies have evaluated whether there is an association between error, boarding, and crowding. Understanding if there is an association is imperative to fully evaluating the risks of providing emergency care during times of increasing crowding and boarding. A greater understanding of the risks and possibilities of errors such as morbidity, mortality, and medicolegal risk could help identify systems changes to address these issues.

### 1.3 Goals of This Investigation

This study evaluates whether ED crowding, boarding, volume, or conditions whereby surge capacity activation criteria (code help status) were met were associated with an error. In particular, we examined whether error occurred based on characteristics of the department at that time.

## 2 Methods

### 2.1 Study Design and Setting

This retrospective cohort study was performed at an urban academic center with a yearly volume of 55,000 patients. The Institutional Review Board at Beth Israel Deaconess Medical Center approved it as exempt. The study complied with the guidelines for conducting retrospective research and reducing bias in emergency medicine chart review studies.^24^

### 2.2 Selection of Participants

We included all patients evaluated in the ED from July 1, 2018 to June 30, 2023, and queried an available database for the presence and absence of an error. The database we used included de-identified information on all patient encounters in the ED, and in particular included operational metrics, presence of an error, and department level conditions. This cohort was consistent with an overall increase in ED volumes (Fig). All protected health information was de-identified at the extraction point, and relevant operational metrics were included during the ED evaluation. This information was obtained through the utilization of our home-grown quality assurance apparatus.

**Figure:**
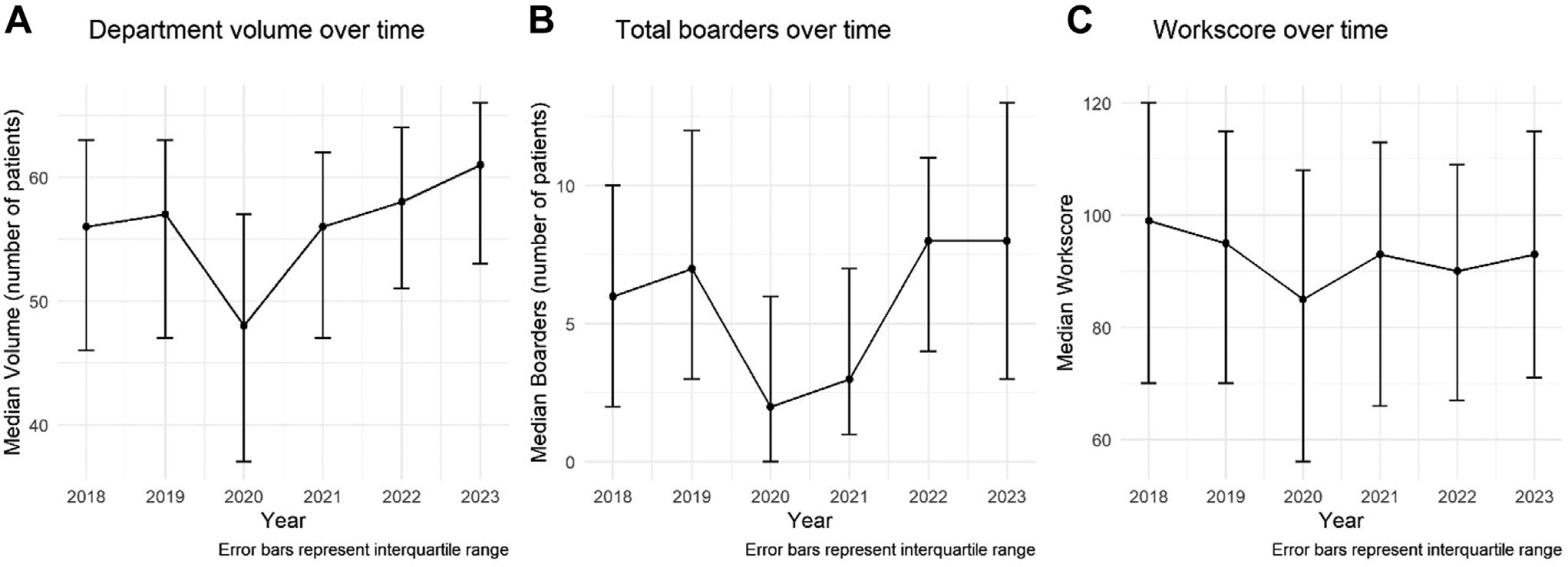
(A) Emergency department volumes, (B) total boarders, and (C) work score from 2018 to 2023.

### 2.3 Measurements

We collected routine demographic data of age and self-reported gender. Operational metrics that were of particular importance were the presence or absence of an error as previously adjudicated by a robust quality assurance apparatus,^23^ measures of crowding (the departmental work score^13^ at the time of arrival, total number of boarders within ED at the time of visit, the total ED volume), and whether the patient was boarding in the ED. Error was broadly defined but was not specified due to the confidential nature of the quality assurance process, nor was any potential harm that reached the patient available. Patients were excluded if they did not have an ESI or a work score.

### 2.4 Outcomes

The primary outcome was the presence of an error as adjudicated by our quality assurance apparatus versus no evidence of an error for that encounter. The key exposures were volume, quartile of work score, code help status (active, alert, or clear), total boarders, and whether the patient was a boarder. Each of these represented either surrogate measures of crowding, direct measures of crowding, or explicit measures of boarding.

### 2.5 Analysis

Patient characteristics were compared based on presence of error. For continuous characteristics, normally distributed data was described using means ± SD and compared using a Student’s *t*-test; nonnormally distributed data were described using medians (IQR) and compared using Wilcoxon ranked-sum tests. Categorical data were described using counts with proportions and compared using a chi-squared or Fisher’s exact test, as appropriate. For hypothesis testing, we performed a Poisson regression model to test the association between the presence of an error and our exposures of interest. We analyzed the data in univariate and controlling for age, gender, and ESI, and calculated incidence-rate ratios (IRR) for our outcome of interest, the presence of an error. All models were tested for evidence of overdispersion and goodness of fit. All analyses were performed using R (R version 4.1. 1 [1]) or Stata 18.2.

## 3 Results

### 3.1 Characteristics of Study Subjects

Over the study period, there were 250,054 ED encounters. Five encounters were excluded due to missing data about ESI or work score, for a final cohort of 250,049. Overall, department volume increased from a low in 2020 to a high in 2023 (Fig). Similar trends were seen in total boarders and the work score (Fig). Less than 1% of all encounters had an error (total of 1238 errors; 0.50 [95% CI: 0.47-0.52]), with an error rate of approximately 500/100,000 (Table 1). Patients with an encounter containing an error were older, had higher acuity, were less likely to be in active code help, were present at a time with lower ED volume, and were more likely to be boarders than those with an encounter without an error (Table 1).

**Table 1:** Baseline demographic and relevant statistical distributions.

Demographics	Error (n = 1238)	No error (n = 248,811)
Age (y), mean±SD	60.4±19.3	54.3±20.7
Female gender, n (%)	632 (51)	133,557 (54)
ESI level, n (%)		
1	130 (11)	11,047 (4)
2	582 (47)	95,706 (38)
3	511 (41)	127,344 (51)
4	14 (1)	14,226 (6)
5	1 (0)	488 (0)
Work score, n (%)		
Q1 (0-66)	347 (28)	63,058 (25)
Q2 (67-92)	308 (25)	62,888 (25)
Q3 (93-113)	308 (25)	62,340 (25)
Q4 (114-194)	275 (22)	60,525 (24)
Code help status (n, %)^a^		
Active	137 (11)	33,590 (14)
Alert	93 (8)	15,281 (6)
Clear	1008 (81)	199.917 (80)
Total boarders, median (IQR)	5 (1, 10)	5 (2, 10)
Total department volume, mean±SD	52.5±11.8	53.9±11.6
Boarders, n (%)^b^	699 (61)	103,839 (46)

ESI, Emergency Severity Index; Q, quartile.

a 23 patients did not have a value for code help status.

b 21,595 do not have information on whether they were a boarder (discharges).

The number of boarders was not significantly associated with the presence of an error; however, whether the patient was a boarder themselves was significantly associated with an increased likelihood of error (adjusted IRR [aIRR]: 1.60 [95% CI: 1.42-1.82]). In univariate, higher acuity was significantly associated with the likelihood of an error (ESI level I, IRR 2.9 [95% CI 2.4-3.5]). When controlling for age, gender, and ESI, the number of boarders was not significantly associated with the presence of an error; however, whether the patient was a boarder themselves was significantly associated with an increased likelihood of error. The total department volume appeared to have a slightly inverse relation with increasing volume portending a slightly less likelihood of an error (aIRR 0.99 [95% CI: 0.99-1.00]). When compared to active status, code help status of clear and alert were both associated with a higher likelihood of error with an aIRR of 1.47 (95% CI: 1.13-1.91) for clear and an aIRR of 1.23 (95% CI: 1.03-1.47). When compared to the first quartile of work score (greater crowding), the fourth quartile of the work score was associated with a reduced likelihood of an error (aIRR 0.81 [95% CI: 0.69-0.95]) (Table 2).

**Table 2:** Unadjusted and adjusted incidence-rate ratios (IRR) for presence of error.

	Unadjusted IRR (95% CI)	Adjusted IRR (95% CI)^a^
Total department volume	0.99 (0.99-1.00)	0.99 (0.99-1.00)
Work score		
Q1 (0-66)	Reference	Reference
Q2 (67-92)	0.89 (0.76-1.04)	0.89 (0.76-1.03)
Q3 (93-113)	0.90 (0.77-1.05)	0.89 (0.76-1.03)
Q4 (114-194)	0.83 (0.71-0.97)	0.81 (0.69-0.95)
Code help status		
Active	Reference	Reference
Alert	1.49 (1.14-1.94)	1.47 (1.13-1.91)
Clear	1.24 (1.03-1.48)	1.23 (1.03-1.47)
Total boarders	0.99 (0.99-1.00)	1.00 (0.99-1.00)
If patient was boarder	1.85 (1.65-2.09)	1.60 (1.42-1.82)

a Adjusted for age, gender, and Emergency Severity Index; Q, quartile.

## 4 Limitations

Our study is a single-center, retrospective analysis of a cohort of patients evaluated in the ED, and as such, has several limitations. Our data capture mechanism does not delineate the time of error occurrence. Therefore, the specific ED volume, number of borders, and crowding metrics are not necessarily specific to the time of the error. Additionally, the number of errors was low; therefore, it is difficult to generalize our results. Most importantly, we could not stratify by type of error or whether an adverse event had occurred, and therefore, it is unclear whether these represent severe adverse events or a minor error without any significant impact on patient care. This information would have provided further context on the types of errors, associated risks, and whether any potential harm reached the patient. Unfortunately, due to the confidential nature of quality assurance, the types of errors were not available. We also do not know how the COVID-19 pandemic impacted data gathering or the adjudication of errors. Volume data was based on half-years for 2018 and 2023, which likely impacted the calculation of the yearly error rate. Additionally, because of the overtaxed nature of staff during crowding, there may be less self-reporting of errors, which may impact error rates or portend selection bias. Lastly, our captured metric of the work score is not universally used, which limits generalizability.

## 5 Discussion

We performed a retrospective cohort study of all patient encounters over the past 5 years to evaluate whether ED crowding or boarding portended a probability of error. This analysis had a low error rate, which did not appear to be associated with increasing crowding. The observed lower rate may be due to utilizing half of a years’ worth of data in 2018 and 2023. Alternatively, there may have been an effect of COVID-19. In our Poisson model, there was a statistically significant relationship between volume and error rate, with the error rate falling with increased volume. There was also an association with higher acuity ESI levels 1 and 2. This is the first such study demonstrating a higher probability of error with higher acuity patients (ESI 1 IRR 2.9 [95% CI: 2.4-3.5], ESI 2 IRR 1.5 [95% CI: 1.3-1.7]). Unexpectedly, our surrogate crowding measures, the work score, ED volume, and code help status active did not demonstrate a higher likelihood of error. Surprisingly, the code help statuses of clear and alert were associated with a higher probability of error. Concerning boarding specifically, with increasing numbers of patients boarding in the ED, there was no higher likelihood of an error. However, the status of being a boarder within the ED was associated with a higher likelihood of error. Curiously, as total department volume increased, there was a slightly decreased probability of an error (aIRR 0.99 [95% CI: 0.99-1.00]).

Previous studies of boarding and crowding focus on patient-level outcomes such as mortality^5^^,^^6^^,^^8^ but do not directly examine errors specific to crowding events or boarding volume. Some studies have demonstrated that less experience and a high ED census are associated with significant errors.^19^ Other studies have shown that high ED crowding was associated with preventable medical errors.^25^, ^26^, ^27^ Interestingly, our study demonstrated that there was not a higher likelihood of error during crowding events, which is in direct contrast to previous literature. Admittedly, the errors in question are not explicitly delineated, and measures of boarding are not directly comparable, so a direct comparison between our findings and those of previous researchers is challenging to define. Regardless, our findings do not appreciate such associations as previously demonstrated.

Our study had unique findings, particularly an inverse relationship between volume and error in our Poisson model. There was a higher likelihood of error when code help status was clear or active, and a substantially higher likelihood of error was observed in patients with higher acuity and older patients. There appears to be a lower likelihood of error with increasing volume. This may be due to a shift structure with significant overlap where fresh teams start as volume increases, or potentially from hypervigilance from ED teams during crowding events. Though we do not have specific information to make suppositions on whether shift hour relates to error likelihood, it could be examined further. Further work in this area could focus on stratifying types of errors with serious adverse events with regard to ESI level, crowding, and boarding.

Lastly, it is well understood that crowding and boarding negatively impact patient care. Our study, while limited, has demonstrated no increased likelihood of error regarding surrogate measures of crowding (ED volume, number of boarders, and code help status active). However, it did demonstrate that higher acuity patients and patients who are boarders themselves are at increased risk of experiencing an error in their care. Our findings suggest that both of these groups are at higher risk of errors and should be carefully monitored.

## Author Contributions

JK, DS, BS, LB, AG, and DC conceived the study, and supervised the conduct of data collection. JK undertook data management. LB and AG provided statistical advice and analyzed the data. JK drafted the manuscript, and all authors contributed substantially to its revision. JK takes responsibility for the paper as a whole.

## Funding and Support

By *JACEP Open* policy, all authors are required to disclose any and all commercial, financial, and other relationships in any way related to the subject of this article as per ICMJE conflict of interest guidelines (see www.icmje.org). The authors have stated that no such relationships exist.

## Conflict of Interest

Dr Kolikof, Dr Shaw, Dr Stenson, Dr Balaji, Dr Grossestreuer, and Dr Chiu report no conflicts of interest.
